# Growth performance and hematological changes in growing pigs treated with *Cordyceps militaris* spent mushroom substrate

**DOI:** 10.14202/vetworld.2020.768-773

**Published:** 2020-04-23

**Authors:** Waewaree Boontiam, Chalong Wachirapakorn, Suchat Wattanachai

**Affiliations:** 1 Department of Animal Science, Faculty of Agriculture, Khon Kaen University, Khon Kaen 40002, Thailand; 2 Department of Surgery and Theriogenology, Faculty of Veterinary Medicine, Khon Kaen University, Khon Kaen 40002, Thailand

**Keywords:** antioxidant capacity, blood metabolites, *Cordyceps militaris* spent substrate, growing pigs, growth performance, immunoglobulins

## Abstract

**Background and Aim:**

This study was aimed to compare the efficacy of dietary *Cordyceps militaris* spent mushroom substrate (CMS) on growth performance, immunity, metabolic profiles, and antioxidant capacity in growing pigs.

**Materials and Methods:**

Seventy-two crossbred growing pigs (Duroc×Landrace×Yorkshire) with an average initial body weight (BW) of 25.78±0.33 kg were allotted into two dietary treatments in six pens (six growing pigs each). Dietary treatments were (i) control and (ii) supplemented group with 2 g/kg CMS.

**Results:**

Growing pigs fed with 2 g/kg CMS showed improvements in final BW (p=0.034) and average daily weight gain (p=0.039). Moreover, there were positive changes in immunoglobulin A (p=0.013), immunoglobulin G (p=0.019), total antioxidant capacity (p=0.001), and glutathione peroxidase activity (p=0.003), whereas decreased leukocyte percentage (p=0.002), cholesterol (p=0.023), and malondialdehyde (MDA) concentrations (p=0.002) were noted in the CMS supplemented treatment. Average daily feed intake, gain-to-feed ratio, glucose, aspartate aminotransferase, triglyceride, high-density lipoprotein, and low-density lipoprotein were unaffected by the treatments.

**Conclusion:**

Supplementation of CMS at 2g/kg of diet increases growth performance, immunoglobulin secretion, and antioxidant capacity, whereas it lowers leukocyte percentage, cholesterol, and MDA concentrations in growing pigs.

## Introduction

*Cordyceps* species are Ascomycetes fungi which invade *Lepidoptera* larvae that have been used as pharmacological food in many countries [1]. These fungi contain various bioactive components, including cordycepin, polysaccharides (β-glucan), and ergosterol [2]. These fungi display immunomodulatory, antioxidant, anti-inflammatory, antibacterial, and antitumor properties [3-5]. Although the advantageous pharmacological functions of *Cordyceps* are well characterized for human health, its application in livestock production is limited due to cost, leading to few studies on the subject.

Koh *et al*. [6] found that *Cordyceps* mycelium can be used as an alternative antibiotic growth promoter to improve weight gain and immunity in broiler chickens. In addition, the inclusion of 1g/kg of fermented *Cordyceps militaris* significantly increased body weight (BW) gain in broiler chickens [7]. In weaning pigs, diets supplemented with 1,000µg/kg fermented *Cordyceps* were shown to promote growth performance and cell-mediated immunity [1]. Therefore, supplementing feed with *C. militaris* spent mushroom substrate (CMS) might prove to be an alternative approach in livestock production not only for improved animal health but also environmental friendliness. It has been documented that the disposal of spent mushroom substrate by industries has increased along with demand [4]. Published reports show that CMS contains several active components such as secondary metabolites, extracellular enzymes, and carbohydrates produced during mycelium and fruiting-body formation [8].

To our knowledge, there are no published reports on using CMS as a feed additive for growing pigs. We hypothesized that the presence of biologically active components in CMS may yield health benefits for growing pigs. Consequently, this research aimed to compare the effects of CMS supplementation on growth performance, immunity, metabolic profiles, and antioxidant capacity in growing pigs.

## Materials and Methods

### Ethical approval

Animal handling protocols were approved in accordance with the Animal Ethics Committee of Khon Kaen University (protocol no. IACUC-KKU103/61).

### Preparation of spent *C. militaris*

The CMS used in this study originated from a Thai rice medium, obtained from a mushroom farm (Samut Prakan, Thailand). After the fruiting bodies of *C. militaris* were harvested, the CMS was subsequently dried in an automatic dry air oven at 50°C for 48h. The CMS samples were subsequently ground and further analyzed for ash, crude protein, ether extract, cordycepin, and gamma-oryzanol contents before supplementation in the feed formulation (Table-1).

**Table-1:** Nutrient and some active compounds in *Cordyceps militaris* spent mushroom substrate.

Composition	Content
Ash (g)	29.8
Crude protein (%)	821.7
Ether extract (%)	138.3
Cordycepin (mg/100 g)	363
Gamma oryzanol (mg/100 g)	48.32

### Animals, experimental design, study period, and management

Seventy-two crossbred growing pigs (Duroc×Landrace×Yorkshire) with an average initial BW of 25.78±0.33kg were divided into two dietary treatments of six pens each, with equally represented gender (six growing pigs each). Dietary treatments, included a control group and a supplemented group, provided with 2g/kg spent *C. militaris* (CMS). Nutrient composition of the basal diet was formulated to meet or exceed the predicted requirement for growing pigs (Table-2) as recommended by the National Research Council [9]. The mash diet was collected in a sealed plastic bag for subsequent sieving through an 80-mesh screen. Representative samples were used for proximate analyses of crude protein (method no.990.03, [10]), ether extract (method no.945.16, AOAC, [10]), and ash (method no.942.05, AOAC, [10]) contents. The gross energy content of the diet was analyzed using a bomb calorimeter (LECO Corporation, USA).

**Table-2:** Feed ingredient and nutrient composition of the basal diet.

Feed ingredient	Amount (%, as fed basis)
Corn	52.33
Soybean meal	31.64
Rice bran	10.00
Rice bran oil	1.71
Dicalcium phosphate	3.24
Salt	0.35
L-lysine monochloride, 98%	0.18
DL-methionine, 98%	0.30
Premi×^1^	0.25
Total	100
Calculated value	
Metabolizable energy (kcal/kg)	3300
Crude protein (%)	18.00
Calcium (%)	0.66
Total phosphorus (%)	0.56
Lysine (%)	1.12
Methionine+Cysteine (%)	0.63
Analyzed composition	
Crude protein (%)	17.37
Ether extract (%)	1.64
Ash (%)	4.67
Gross energy, MJ/kg	16.34

1 Supplied (per kilogram diet): Vitamin A as retinol, 8400 IU; Vitamin D3, 945 IU; Vitamin E, 0.0126 g; Vitamin K, 0.0021 g; Vitamin B1 (thiamine), 0.0011 g; Vitamin B2 (riboflavin), 0.0022 g; Vitamin B6 (pyridoxine), 0.0016 g; Vitamin B12 (cyanocobalamin), 0.02 mg; nicotinic acid, 0.0126 g; pantothenic acid, 0.063 g; folic acid, 0.0053 g; biotin, 0.0315 mg; choline, 0.175g; copper as CuSO_4_, 0.126 g; iron as FeSO_4_, 0.105 g; manganese, 0.021 g; cobalt, 0.0007 g; iodine, 0.0007 g; selenium as Na_2_SeO_3_, 0.00007 g

All experimental pigs were housed in open pens, during January-February 2019 in a 6-week feeding trial. The housing temperature was controlled manually by an electric fan when the temperature rose above 28°C. Daily temperatures ranged from 23 to 25°C (09:00 pm to 06:00 am) and 27 to 35°C (09:00 am to 05:00 pm). Experimental pigs were raised in a 4m wide x 5m long pen space (at a stocking density of 3.33 m^2^/pig), equipped with an automatic drinking water dispenser and a self-feeder to ensure pigs had *ad libitum* access to feed and water. The experimental diet was fed 3times daily at 06:00, 12:00, and 17:00 throughout the study.

### Growth performance

Each individual pigs’ BW was assessed at the beginning and termination of the experiment. Total feed supplied, spilled feed, and leftover feed presence were recorded on a per pen basis and used to adjust feed intake. The collected data were further used to calculate average daily gain (ADG), average daily feed intake (ADFI), and the gain-to-feed (G:F) ratio. The ADG and ADFI calculations were determined by dividing total weight gain and total feed intake in each pen by the number of pig-feeding days. The G:F ratio was calculated for each pen by dividing the ADG by ADFI.

### Diarrheal score

The diarrhea incidence of the growing pigs in each pen was monitored daily at 06:00 for 3weeks of the experimental period. The diarrheal score was assessed visually, on a per pen basis, by two observers and the average values were recorded for fecal consistent score. Scores were given using a 5-point scale (1=hard feces, 2=no diarrhea occurrence with normal feces and consistent formation, 3=mild diarrhea with soft and partially formed feces, 4=moderate diarrhea with loose and semiliquid feces, and 5=severe diarrhea with watery feces). The percentage of diarrheal occurrence was calculated as (total number of pigs with diarrhea in each treatment/[total number of pigs in each treatment×experimental day]) x 100 [11].

### Blood collection and analyses

Blood collection was performed after a 12h fast on day 42 of the experiment. Twenty-four growing pigs (12 barrows and 12 gilts), from each treatment, were randomly chosen to collect blood samples using disposable syringes with needles. Each 8mL sample was carefully transferred to a vacuum tube (Becton Dickinson Vacutainer Systems, Franklin Lakes, NJ). Samples were placed at room temperature for 2h following collection, after which sera were isolated by centrifuging at 3000×g for 15min. The sera were used to assess hematological values.

Immunoglobulin concentrations were quantified, with specific porcine immunoglobulin A (IgA, no. A100-102, immunoglobulin G [IgG], no.100-104), using ELISA Quantitation kits (Bethyl Laboratories, Inc., Montgomery, TX, USA). The number of white blood cells present in samples was analyzed using a blood analyzer (Diamond Diagnostic Inc., Holliston, USA). Leukocyte percentage was calculated per leukocyte by comparing the amount of each leukocyte relative to the total amount of leukocyte present in each sample. Metabolic profiles for glucose (Glucose Hexokinase Kit, Roche Diagnostics), aspartate aminotransferase (AST), triglyceride, cholesterol, high-density lipoprotein (HDL), and low-density lipoprotein (LDL) were determined using an autoanalyzer. Concentrations of total antioxidant capacity (TAC) and glutathione peroxidase (GSH-Px) were measured using commercial kit tests (Nanjing Jiancheng Bioengineering Institute, Nanjing, China), and detected values were tested using a spectrophotometer. Each sample was analyzed in triplicate to minimize variation.

### Statistical analyses

All data are presented as mean±standard error of differences. Growth performance, diarrheal incidence, and all metabolic measurement data were analyzed using Student’s t-test in the statistical software (SAS University Edition) with each pen or individual pig as the experimental unit. The statistical difference between treatments was considered at the probability cutoff of 0.05 or 0.01 where applicable.

## Results

### Growth performance and diarrheal occurrence

The supplementation of 2g/kg CMS contributed to significant improvements in final BW (p=0.034) and ADG (p=0.039) of growing pigs on day 42 (Table-3). The ADFI (p=0.078) and G:F ratio (p=0.066) were slightly increased in CMS supplemented treatment in comparison to the control. However, the results obtained for ADFI, G:F ratio, and diarrheal occurrence were unaffected by dietary treatment.

**Table-3:** Growth performance and diarrheal incidence in growing pigs fed *Cordyceps militaris* spent mushroom substrate^1^.

Criteria	Control	*Cordyceps militaris* spent	SED	p-value
Initial BW, kg	25.86	25.69	0.333	0.809
Final BW, kg	49.52	58.59	2.229	0.034
ADG, g	563.31	783.42	55.454	0.039
ADFI, kg	1793	1924	37.236	0.078
G: F ratio	0.314	0.407	0.025	0.066
Diarrheal incidence (%)	0.15	0.12	0.032	0.632

BW=Body weight, ADG=Average daily gain; ADFI=Average daily feed intake, and G: F=Gain-to-feed ratio.

1 A total of 72 growing pigs had an average initial BW of 25.78±0.33 kg and a final BW of 54.06±2.23 kg, SED=Standard error of the differences

### Immunoglobulins and leukocytes

There was no significant difference in leukocyte percentage between the treatments (Table-4), except for the lymphocyte percentage (p=0.002). In addition, IgA (IgA; p=0.013) and IgG (IgG, p=0.019) secretory levels were positively affected by CMS dietary supplementation treatments.

**Table-4:** Immunoglobulins and leukocyte percentage in growing pigs fed *Cordyceps militaris* spent mushroom substrate^1^.

Criteria	Control	*Cordyceps militaris* spent	SED	p-value
Immunoglobulins				
IgA (mg/ml)	1.76	2.94	0.260	0.013
IgG (mg/ml)	5.94	8.18	0.511	0.019
Leukocytes				
Lymphocytes (%)	74.34	46.67	5.292	0.002
Neutrophils (%)	39.38	33.83	3.238	0.417
Monocytes (%)	1.21	1.26	0.190	0.916
Eosinophils (%)	0.46	0.41	0.091	0.773

1 Each mean represents 6 pens of 12 growing pigs (n=24), SED=Standard error of the differences IgG=Immunoglobulin G, IgA=Immunoglobulin A

### Blood metabolic profiles

Throughout the entire experiment, the concentrations of glucose, AST, triglyceride, HDL, and LDL were not affected by spent *C. militaris* supplementation (Table-5). However, cholesterol concentrations were significantly lower in the CMS supplemented group when compared to the control group.

**Table-5:** Metabolic blood profiles in growing pigs fed *Cordyceps militaris* spent mushroom substrate^1^.

Criteria	Control	*Cordyceps militaris* spent	SED	p-value
Glucose (mg/dL)	115.81	128.63	11.650	0.606
AST (U/L)	51.34	50.17	4.934	0.912
Triglyceride (mg/dL)	115.41	91.62	12.204	0.354
Cholesterol (mg/dL)	125.51	98.72	6.233	0.023
HDL (mg/dL)	34.19	45.03	4.091	0.198
LDL (mg/dL)	39.83	32.19	4.135	0.381

AST=Aspartate aminotransferase, HDL=High.density lipoprotein, and LDL=Low.density lipoprotein.

1 Each mean represents 6 pens of 12 growing pigs (n=24). SED=Standard error of the differences

### Antioxidant capacity

The growing pigs fed 2g/kg CMS had greater concentrations of TAC (p=0.001) and GSH-Px (p=0.003), whereas they had lower malondialdehyde (MDA) concentrations (p=0.002) than those fed the control diet (Table-6).

**Table-6:** Antioxidant capacity in growing pigs fed *Cordyceps militaris* spent mushroom substrate^1^.

Criteria	Control	*Cordyceps militaris* spent	SED	p-value
Total antioxidant capacity (U/ml)	1.03	1.86	0.142	0.001
Glutathione peroxidase (U/ml)	568.28	951.74	0.691	0.003
Malondialdehyde (nm/ml)*	7.37	3.71	75.032	0.002

1 Each mean represents 6 pens of 12 growing pigs (n=24), SED=Standard error of the differences

## Discussion

Alternatives for antibiotic growth promoters have been introduced to livestock production worldwide. *Cordyceps* species and it’s constitutes can improve growth performance of healthy pigs by stimulating the immune system and beneficially affecting intestinal microbiota [1,7]. In this study, greater final BW and ADG exhibited by pigs fed a diet containing 2g/kg CMS may have been related to the improvements in immunoglobulin and lymphocyte secretions. CMS supplementation is expected to enhance the health status of growing pigs, which subsequently improves their growth rate. Our findings are consistent with Cheng *et al*. [1], who observed that fermented *C. militaris* contributed to the enhancement of weaned pigs’ performance, as had been detected in the previous reports [6,7]. The obtained data suggest that CMS supplementation, in the diet of growing pigs, should not have any detrimental effects on their growth performance and feed efficiency.

Serum immunoglobulins are an important indicator for defining humoral immunity in pigs. IgA and IgG play a key role in defense against pathogenic invasion, and thus, changes in these protein levels can affect a pig growth performance and immunity [12]. Our data showed that growing pigs, receiving a CMS supplemented diet, exhibited greater secretions of IgA and IgG, whereas they had a decreased lymphocyte percentage compared to pigs fed a control diet. The previous studies, which are in line with our findings, have shown that bioactive cordycepin, produced by *C. militaris*, functions on immune stimulation [1,13]. An explanation for this stimulation may be that CMS, originating from a Thai rice medium, contains a high amount of γ-oryzanol, which has been shown to have a potential effect on immunity, by activating IgA production [14,15]. In addition, the presence of γ-oryzanol has been shown to activate cytokine secretion and thus reduce mucosal inflammation associated with *Salmonella* infections [15,16]. This implies that an increase in macrophage function may protect tissue from damage, in the growing pigs fed CMS, during an invasion of pathogenic bacteria.

Changes in metabolic profiles can be used as an effective indicator in detecting animal health status. This research observed that the concentration of AST was unaffected by dietary treatments, indicating that CMS supplementation was not harmful to the growing pigs’ health. However, cholesterol concentration was decreased in pigs receiving CMS. The underlying mechanism in livestock animals has not been established, but a positive effect was shown in hamsters. Guo *et al*. [17] demonstrated that daily administration of cordycepin positively reduced the accumulations of total cholesterol, triglyceride, and LDL. This enzyme plays a key role in decreasing total cholesterol by inhibiting the activity of glycerol-3-phosphate acyltransferase and HMG-CoA reductase, which further contribute to the regulation of cholesterol synthesis [18]. Lai *et al*. [19] also demonstrated a significant reduction in total cholesterol concentration by inhibiting lipid absorption from intestine. Our results are in contrast to Cheng *et al*. [1], who observed that the metabolic profile of triglyceride was changed by the fermented *C. militaris*, whereas no significant effect was found for cholesterol concentration. This discrepancy might be attributed to the dosage of cordycepin content, the source of mycelium *C. militaris* media, dietary composition, and the growth stage of pigs. This suggests that controlling hyperlipidemia by the dietary supplementation of CMS for growing pigs is a beneficial approach.

Imbalances of the oxidant-antioxidant system have been reported to increase oxidative stress [20]. GSH-Px is an important antioxidant enzyme that is normally found inside cells. It functions by converting hydrogen peroxide to water and simulates oxidized glutathione through glutathione disulfide [21]. Decreases in GSH-Px and TAC lead to the activation of reactive oxygen species, increasing cell susceptibility to increased MDA production that further causes DNA damage [22]. This study showed that growing pigs fed with 2g/kg CMS could resist oxidative stress through increased TAC and GSH-Px production, while displaying decreased MDA concentration. Our observations are consistent with Ramesh *et al*. [23], who observed that cordycepin (3’-deoxyadenosine) elevated GSH-Px and decreased MDA concentration. These potential effects may account for the high amount of cordycepin and γ-oryzanol present in the CMS of *C. militaris* when cultured on a rice medium. The previous findings have shown that γ-oryzanol is a stronger antioxidant than Vitamin E [24]. It might be possible that both active compounds presented in CMS are effective for balancing antioxidant status and preventing lipid peroxidation in growing pigs.

## Conclusion

Growing pigs fed a diet supplemented with 2g/kg CMS showed greater improvements in BW, ADG, immunoglobulin secretions, and antioxidant status; they showed decreased lymphocyte percentage, cholesterol, and MDA concentrations without impairing hepatic function.

## Authors’ Contributions

WB contributed to the main conceptual design, performed experiments, and drafted the manuscript. CW contributed by supervising and contributing to the finding of this research. SW supervised animal handing throughout the entire project. All authors read and approved the final manuscript.
